# Double-Microcatheter Technique through Tortuous Anatomy for Coil Embolization of a Saccular $plenic Aneurysm: a Technical Report

**Published:** 2020-02-20

**Authors:** M Panagrosso, C De Gregorio, A Peluso, P Venetucci, G Buono, UM Bracale

**Affiliations:** 1 Department of Public Health, Unit of Vascular Surgery, University Federico II of Naples, Italy; 2 Department of Radiology, University Federico II of Naples, Italy

**Keywords:** visceral aneurysm, endovascular technique, embolization

## Abstract

We report on a case of an asymptomatic splenic artery aneurysm (SAA) with a large neck in a 53-year-old female with an extreme vessel tortuosity which was treated with a Double Microcatheter Technique. This endovascular procedure consists of embolization of the aneurysm using detachable coils with no application of any glue, stent or balloon.

At the end of procedure, no complications occurred. At the three-month follow-up an MRI showed the aneurysm’s complete exclusion and patency of the splenic artery.

## I. INTRODUCTION

Splenic artery aneurysms (SAAs) are the third most frequent aneurysms in the abdomen after aneurysms of the aorta and iliac arteries and are the most frequent visceral aneurysms with a reported prevalence of 0.8% on arteriography and 0.04–0.10% at autopsy 1.

Rupture remains the most feared complication with incidence ranging from 2.3% to 18% and a mortality rate ranging from 20 to 100% 2. SAAs are predominant in females with a rate of 4:1, mostly located in the mid to distal third of the splenic artery. The rapid evolution and improvements in cross-sectional imaging will undoubtedly increase the prevalence of SAAs in the future and may help in treating them earlier in their natural history 3.

Size is the first indication for treatment of asymptomatic SAA considering that a diameter greater than 20 mm is related to a high risk of rupture 4–6. Percutaneous transcatheter embolization of SAAs with coils is widely accepted as the first line of treatment due to its safety, low mortality rate and because it provides adequate short- and long-term results 7,8. Anyway in cases of wide-neck aneurysms the risk of coil migration increases significantly. To reduce the rate of this occurrence, different kinds of endovascular techniques such as balloon and bare stenting-assisted have been introduced.

In this report we present the case of a saccular SAA successfully treated with coil embolization using the double-microcatheter technique; a further endovascular approach in treating SAAs in patients with tortuous vessel anatomy.

## II. CASE REPORT

**Figure 1:**
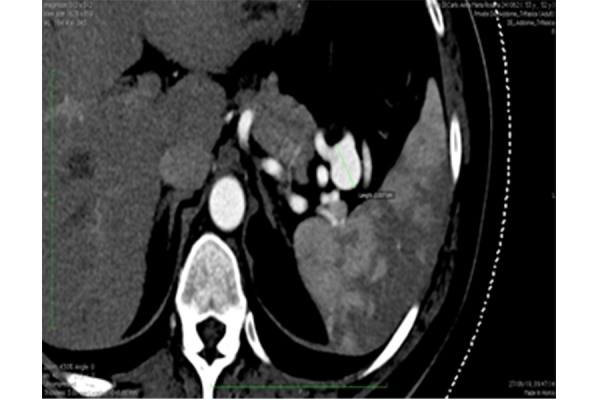
Ct-Scan

A 53-year-old mother of two children with a family history of lung carcinoma and affected with hypertension, dyslipidemia and sideropenic anemia, underwent a duplex scan to investigate a chronic biliary micro-lithiasis. The exam detected an abdominal lesion suspected of adrenal adenoma. The patient was submitted to a CT-scan that revealed a saccular aneurysm (maximum diameter 20 mm x 19 mm, neck diameter 9 mm) in the medium-distal part of the splenic artery which also presented an extremely tortuous course (figure 1). Given the aneurysm’s large neck and the relatively young age of the patient, she was admitted to our unit for a therapeutic procedure with an advanced endovascular approach for which she gave her written informed consent before procedure. At hospital admission, her clinical conditions were good and the electrocardiogram and blood tests did not show any alteration. She was transferred to the angio-suite in order to perform the procedure.

Under local anesthesia a 7 Fr vascular sheath was positioned in the right common femoral artery. A 5-Fr Simmons 1 catheter (Glidecath) was advanced through the celiac artery on a standard guidewire (0.035′, Terumo) and selective catheterization of the splenic artery was carried out.

**Figure 2:**
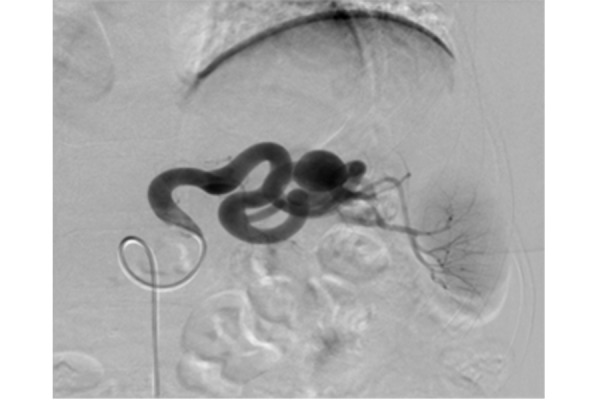
Angiographic control

The following angiographic control confirmed the extreme tortuosity of the splenic artery and the saccular dilatation in its medium-distal part (figure 2). The Simmons 1 was changed in favour of a Mach 1 RDC 7 Fr guide-catheter (Boston Scientific) to advance into the splenic artery near the aneurysm.

**Figure 3:**
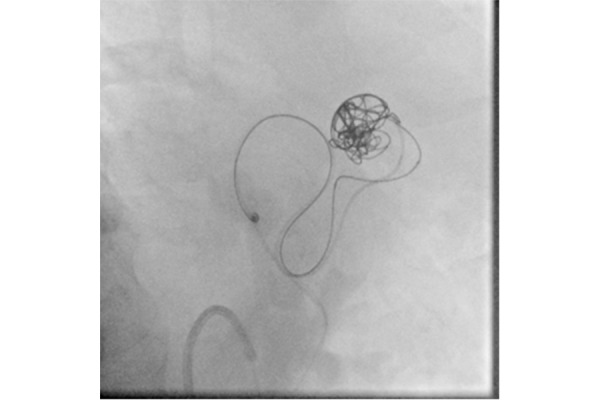
Double microcatheter technique

It was decided to perform the double microcatheter technique to avoid coil migration and therefore 2 microcatheters were inserted through the guiding-catheter and positioned up to the proximal and distal part of the neck of the aneurysm, respectively. (figure 3).

**Figure 4:**
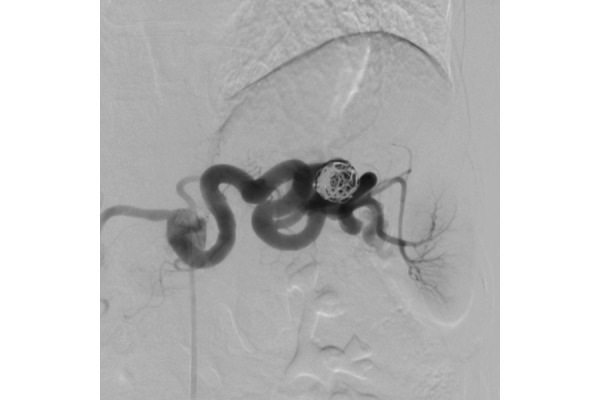
Complete aneurysm exclusion and patency of the splenic artery

Coil packing was performed using detachable coils, alternately released through the 2 microcatheters to obtain a safe and accurate placement. Four coils (Helix ev3 Axium 3d 20mm × 50 cm and 18 mm × 40 cm, Helix ev3 Axium 16 mm × 40 cm and 14 mm × 40 cm) were used overall. The last angiogram showed complete aneurysm exclusion and patency of the splenic artery (figure 4). The patient was discharged the day after this uncomplicated procedure.

**Figure 5:**
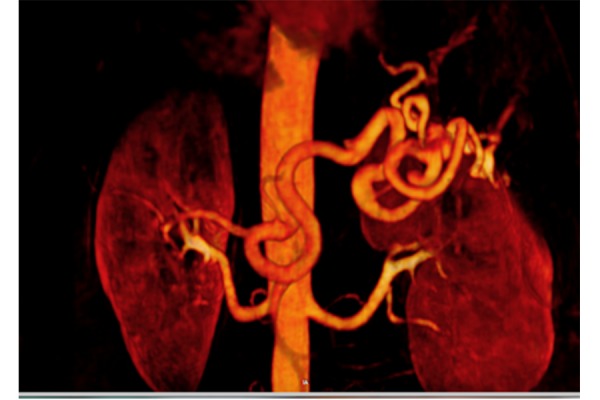
MRI at 3 months

After three months an MRI was given showing good coil positioning with complete exclusion of the aneurysm, total splenic artery patency and no evidence of splenic ischemic lesions or infarctions (figure 5). The patient was doing well 6 months after the procedure.

## III. DISCUSSION

Visceral artery aneurysms represent a rare entity but are a serious and potentially lethal condition when rupture occurs. Treatment options include standard surgery and endovascular embolization with either coils or glue or both or aneurysm exclusion with a covered stent.

The surgical approach has shown excellent long-term results but is still burdened by higher rates of periprocedural morbidity and mortality, longer operative times and length of stay 9,10.

The endovascular treatment was initially reserved for high risk patients or in emergency conditions when rupture occurs but nowadays this approach is first choice in many centers due to technical success and lower mortality comparable with traditional open repair procedure. Furthermore, the endovascular technique is less invasive and associated with a shorter length of stay with a mean hospital stay of 3.8 vs 12 days 8,9.

The two most common endovascular techniques are transcatheter embolization and covered stenting. Transcatheter embolization is currently considered a first choice for saccular aneurysms 11. Ikeda et al. report on their experience with transcatheter coil embolization for patients with saccular and proximal visceral artery aneurysms that may only be treated by coil-packing using coil and/or cyanoacrylate or thrombin, engaging the aneurysm neck and limiting embolization to the sac preserving native arterial circulation 5.

The use of covered stenting for SAAs exclusion is widely consolidated offering the potential benefit of maintaining splenic perfusion and eliminating risk of rupture however it has been reported to not work 30% of the time as it requires a favorable anatomy for a correct positioning 6,12.

Recently the use of stent-assisted coil embolization or multilayer flow modulator stents has extended endovascular treatment to multiple types of complex aneurysms 13,14. However the use of balloon or bare stents placed across the aneurysm neck to prevent coil migration may involve some risks such as dissection and thromboembolic phenomena, distal ischemic events and aneurysmal rupture due to compressive forces generated by balloon inflation. The main failures of the endovascular approach include persistent aneurysm perfusion, recanalization and coil migration. A reperfusion rate of 10.3% in the first month has been observed 15.

Failures may be related to technical difficulties in catheterizing the aneurysm neck resulting in intra-procedural dissection or rupture and may more frequently occur in treatment of larger aneurysms, a large neck size and flow turbulence which can lead to unstable coils and incomplete sac packing 8.

The extreme tortuosity of the splenic artery which was the main feature in our case led us to perform the SAAs exclusion using the double microcatheter technique which could be considered an evolution on the standard transcatheter embolization. This technique is widely used for wide-necked and irregular-shaped intracranial aneurysms 16,17 but it also linked to good outcomes even for the treatment of visceral aneurysms with severe tortuosity of the native artery 7.

A limit to this approach consists of the angiographic acquisitions which could be difficult through a 4Fr–5Fr guiding sheath because of the smaller internal space due to the use of microcatheters. To avoid this problem we used a 7Fr guiding sheath.

A recent report 7, describes several advantages and tricks for the success of this technique such as the ability to work on extremely tortuous arteries due to the size and manageability of the devices, the possibility to recapture the first coil if the second one displaces itself and repeated coil placement attempts until a good frame is achieved.

We chose to follow up the patient with a contrast enhanced MRI because of its reported usefulness in post-treated intracranial aneurysms and pulmonary arteriovenous malformations. Kawai et al. reported that the advantages of MRI compared to contrast-enhanced and/or unenhanced CTs were: 1) less susceptibility to metallic artefacts from platinum coils, 2) detectability of dynamic blood flow at a high temporal resolution, and 3) no radiation exposure 18,19. Based upon our successful outcome we can conclude that the double-microcatheter technique represents a good choice in treating SAAs when native artery shows a severe tortuosity course with a low risk of coil migration, even in the case of large necks, and with no need to use any glue, stents or balloons.
